# Accelerating Digitalization in Healthcare with the InSilicoTrials Cloud-Based Platform: Four Use Cases

**DOI:** 10.1007/s10439-022-03052-6

**Published:** 2022-09-08

**Authors:** Chiara Nicolò, Fianne Sips, Cristina Vaghi, Alessia Baretta, Vincenzo Carbone, Luca Emili, Roberta Bursi

**Affiliations:** 1 InSilicoTrials Technologies S.P.A, Riva Grumula 2, 34123 Trieste, Italy; 2 InSilicoTrials Technologies B.V., Bruistensingel 130, 5232 AC ’s Hertogenbosch, The Netherlands

**Keywords:** Virtual patient, Virtual cohorts, *In Silico* trials, Modeling and simulation, Machine learning

## Abstract

The use of *in silico* trials is expected to play an increasingly important role in the development and regulatory evaluation of new medical products. Among the advantages that *in silico* approaches offer, is that they permit testing of drug candidates and new medical devices using virtual patients or computational emulations of preclinical experiments, allowing to refine, reduce or even replace time-consuming and costly benchtop/*in vitro*/*ex vivo* experiments as well as the involvement of animals and humans in *in vivo* studies. To facilitate and widen the adoption of *in silico* trials, InSilicoTrials Technologies has developed a cloud-based platform, hosting healthcare simulation tools for different bench, preclinical and clinical evaluations, and for diverse disease areas. This paper discusses four use cases of *in silico* trials performed using the InSilicoTrials.com platform. The first application illustrates how *in silico* approaches can improve the early preclinical assessment of drug-induced cardiotoxicity risks. The second use case is a virtual reproduction of a bench test for the safety assessment of transcatheter heart valve substitutes. The third and fourth use cases are examples of virtual patients generation to evaluate treatment effects in multiple sclerosis and prostate cancer patients, respectively.

## Introduction

Modeling and simulation (M&S) approaches are playing an increasingly important role in the design and development of new medical products for assessment of their safety and/or efficacy, and furthermore in supporting regulatory submission and decisions. *In silico* techniques can provide predictions about toxicity, efficacy, optimal dosing strategies and study design, and are currently employed by pharmaceutical companies to guide the different stages of the drug development process, from discovery through the preclinical and clinical phases, drug registration as well as to leverage post-market real-world data.^20^ Similarly, *in silico* tools can be applied to different phases of medical device design, from concept through verification and certification, to make predictions about mechanical performance, durability and resistance, deployment effects and interaction with human anatomy.^8^

One of the powerful advantages of simulation techniques is that drug candidates or new devices can be tested on virtual patients within *in silico* clinical trials. This allows to investigate several “what if” scenarios while minimizing interventions on real individuals, by far ethically sounder than traditional “trial and error” approaches. A virtual patient is a computational model representing the anatomy and/or (patho-)physiology of interest, as well as the pharmacokinetic (PK) and pharmacodynamic (PD) properties of pharmaceutical compounds or the interaction with the medical device. Virtual patients are generally created by directly measuring or estimating the model parameters from experimental or clinical data, or based on literature knowledge, so that simulation outputs are physiologically plausible, i.e., they can reproduce processes occurring in realistic human subjects.

A virtual patient cohort consists of multiple virtual patients with a common model structure but distinct parameter values, which allow to account for inter-patient variabilities in anatomy, physiology, and/or PK/PD properties, depending on the specific context of use for the model.

Different strategies can be applied to generate virtual cohorts. A first method consists of sampling the model parameters from inferred parameter distributions. This approach is commonly applied in semi-mechanistic PK/PD models, where population distributions of the model parameters are usually inferred under the mixed effects modeling framework.^16^ More computationally intensive quantitative system pharmacology (QSP) models require different sampling techniques, such as random generation of the model parameters in conjunction with verification of acceptance criteria, where parameter sets are accepted only if the resulting model output falls within observed physiological ranges.^1^

For medical device applications, human anatomy is usually reconstructed by medical image segmentation, and then combined with other numerical parameters (e.g., tissue mechanical, thermal and electrical properties) and boundary conditions (e.g., external forces during daily living activities, blood flows and pressures at geometry inlets and outlets) which represent the input for the mechanistic computational model.^24^ Additional methods such as principal component analysis, statistical shape modeling and population analysis are then used to generate virtual populations starting from real clinical datasets.^26^

*In silico* trials methodologies are not restricted to clinical contexts, being also applied to conduct virtual preclinical studies, allowing to refine, reduce and potentially replace benchtop, *in vitro* and *ex vivo* experiments as well as *in vivo* studies in animals.^36^ For example, the model developed by Paci *et al.*,^22^ permits the virtual emulation of the cardiac safety *in vitro* test on human induced pluripotent stem cell-derived cardiomyocytes (hiPSC-CMs). Similarly*, in silico* reproduction of existing standard testing allows to refine and reduce the number of physical experiments to be performed.^15^ Recently, standard practices for finite element analyses have been codified to virtually test medical devices.^2^

**Figure 1:**
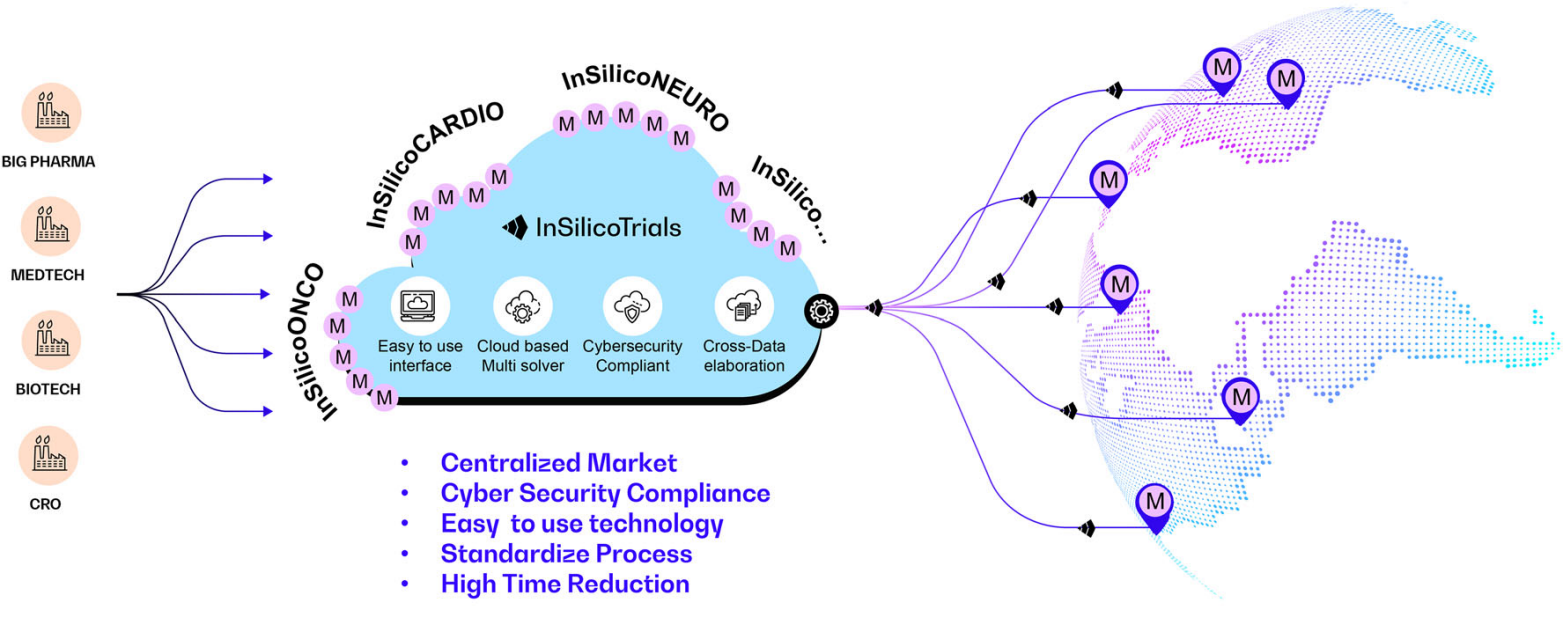
Schematic representation of the InSilicoTrials.com collaborative model. Through a cloud-based platform, InSilicoTrials.com provides Pharma and MedTech companies with computational models developed by internationally recognized universities and research centers. Computational models are integrated into user-friendly simplified workflows, which user can access with the highest level of data security in a pay-per-use way.

*In silico* trials methodologies are nowadays encouraged by regulatory authorities such as the US Food and Drug Administration^10^ and the European Medicines Agency^7^, and included in the new EU Medical Device Regulation.^28^ Nevertheless, the requirements for technical computational skills and the need of extensive verification, validation, and credibility assessment, along with substantial investment in software licenses and IT infrastructure, have considerably limited their adoption to M&S consulting companies and large pharmaceutical and MedTech companies, while remaining an insurmountable challenge for small and medium-sized enterprises. To disrupt the M&S adoption barrier, we have developed an easy-to-use cloud-based commercial platform, where advanced healthcare simulation solutions, developed by internationally recognized universities and research centers, are made available, often for the first time, to medical device and pharmaceutical companies to accelerate time and to reduce costs of their research and development processes (Fig. 1).

The purpose of this paper is to discuss a selection of *in silico* trial applications for drug and device development through the illustration of four use cases built by the authors using simulation solutions available on the InSilicoTrials.com platform.

## Materials and Methods

InSilicoTrials.com is a unique web-based cloud-based platform offering advanced M&S tools to perform *in silico* trials analyses. The platform is proposed as a pay-per-use Software as a Service solution. It integrates various original computational solutions (e.g., ordinary differential equations-based semi-mechanistic population PK/PD models, mechanistic QSP agent-based models, partial differential equations-based finite element methods and computational fluid dynamics, AI-based models, as well as hybrid methodologies) provided by the research community and integrated into user-friendly online applications. The different computational solutions are organized by therapeutic areas (including cardiology, oncology, neurology, nephrology, orthopedics) and collected into suites of complementary applications (e.g., “Drug Safety Suite” for torsadogenic risk assessment). The integrated M&S workflows on the cloud platform allow users to upload their own input data, automatically set up trial scenarios, run simulations and process outcome. Finally, the user has the possibility to export the outcome of the simulations in an automatically formatted report.

The proposed platform embeds a variety of programming languages (e.g., C++, Python, R, Matlab) and simulation engines (e.g., NONMEM, ANSYS, Abaqus, CodeASTER, OpenFOAM) with no direct access to the solvers by the user. It is built on the Microsoft Azure cloud environment, in compliance with the highest standards of security and privacy (amongst others HIPAA Privacy and Security Rules; ISO/IEC 9001, 20000, 22301, 27017, 27018 and 27001; FDA 21 CFR Part 11 (GxP); Protection Directive 95/46/EC), protecting the IP of model providers against the downloading, copying, and changing of their models, while providing a safe environment for users to manage their own data.

## Results

This section presents four use cases of *in silico* trials built by the authors using simulation tools available on the InSilicoTrials.com platform. The first application illustrates how *in silico* approaches based on virtual human myocardial cells can complement *in vitro* tests to improve the early assessment of drug-induced cardiotoxicity risks. The second use case is a virtual emulation of a bench standard test to characterize the mechanical properties of transcatheter heart valve replacements. The third and fourth use cases are examples of *in silico* approaches to simulate clinical trials and evaluate treatment effects in relapsing remitting multiple sclerosis (RRMS) and prostate cancer (PCa) patients, respectively.

### Early Assessment of Drug-Induced Proarrhythmic Risk Using *In Silico* Reconstructions of Human Myocardial Cells

Torsade de pointes (TdP) is a life-threatening cardiac arrhythmia that has led to the withdrawal of many drugs from the market. Although the adoption of the preclinical and clinical safety guidelines (ICH S7B, ICH E14) has been effective in preventing torsadogenic compounds from reaching the market, their lack of specificity might have stopped a number of safe compounds from further development.^11^

In 2013 the Comprehensive *in vitro* Proarrhythmia Assay (CiPA) initiative was established to improve the accuracy of torsadogenic risk predictions by using—in an evaluation phase—*in vitro* experiments of drug effects on multiple ion channels incorporated into *in silico* reconstructions of the action potential of human cardiomyocytes, and—in a subsequent validation phase—*in vitro* assessment of drug effects on multiple ion channels in hiPSC-CMs to detect any missed torsadogenic effects.^4^ In 2020 the ICH S7B/E14 Q&As was released to update the previous safety guidelines in light of the CiPA initiative.

In line with the CiPA paradigm, we have developed two online applications to complement the *in vitro* early screening of a compound’s proarrhythmic risk: QT/TdP Risk Screen and STrhiPS.

#### QT/TdP Risk Screen

QT/TdP Risk Screen (qttdp.insilicocardio.com) is a web-based implementation of an *in silico* risk classifier for the assessment of drug proarrhythmicity.^18^ QT/TdP Risk Screen was built using a machine learning tool, based on cellular simulations of the O’Hara *et al.* human ventricular action potential model^21^ and existing pharmacological data (IC_50_ and effective free therapeutic plasma concentration) for 109 drugs of known torsadogenic risk from the CredibleMeds database.^37^ Cellular simulations were used to derive four *in silico* arrhythmogenic biomarkers, which were then combined into ensemble decision tree classifiers. The final classifier combines biomarkers calculated taking into account effects on the four most prominent ion currents—*I*_Kr_ (fast component delayed rectifier current), *I*_Ks_ (slow component delayed rectified current), *I*_CaL_ (L-type calcium current) and *I*_NaL_ (late sodium current)—and achieved accuracy, sensitivity and specificity values of 94.5%, 94% and 95%, respectively,^18^ in cross-validation on the CredibleMeds database.

Prediction of a compound’s proarrhythmic potential with the QT/TdP Risk Screen tool requires the *in vitro* IC_50_ and (optionally) Hill coefficient values for up to the four ion currents, and the compound’s test concentration values for which the proarrhythmic risk needs to be assessed. The tool’s outcome is the compound classification in one of the possible clinical risk categories: unsafe, probably unsafe, probably safe, or safe.

#### STrhiPS

STrhiPS (Safety Trials on Human Induced Pluripotent Stem Cells, strhips.insilicocardio.com) enables to conduct *in silico* safety trials on a virtual population of hiPSC-CMs, accounting for drug effects on the seven main ion currents indicated by the CiPA initiative.^11^ The tool is based on a state-of-the-art hiPSC-CM action potential model integrating multiple current-drug interactions.^22^ The cellular model was validated against current blocking experiments and was further extended to a population of 1774 *in silico* cell models, calibrated by using a multi-objective genetic algorithm together with optically recorded action potential and Ca^2+^ transient data.^22^

Trial simulations with the STrhiPS tool require the number of hiPSC-CM cells to be simulated (up to 1774 cells), the *in vitro* IC_50_ and (optional) Hill coefficient values for up to seven ion currents (*I*_Kr_, *I*_Ks_, *I*_CaL_, *I*_Na_, *I*_NaL_, *I*_to_, *I*_K1_) and the concentration values for which the compound needs to be tested. Detailed simulation results are provided to the user both in terms of arrhythmogenic biomarkers distributions for the virtual cell population and in terms of plots of membrane potential traces in each virtual cell.

#### *In silico* Predictions of the Torsadogenic Potential for Eight Example Compounds

**Figure 2:**
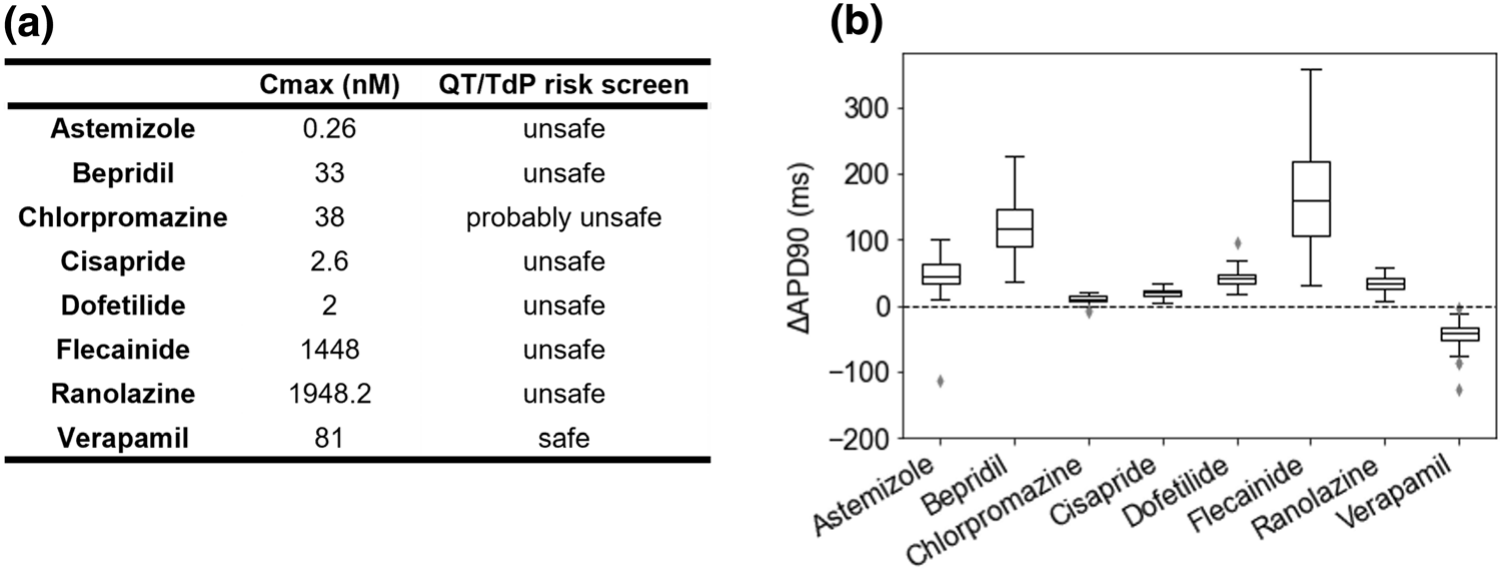
QT/TdP Risk Screen and STrhiPS drug-induced cardiotoxicity predictions. (a) QT/TdP Risk Screen clinical risk classification of a dataset of known drugs at therapeutic concentration *C*_max_. (b) Difference in the action potential duration at 90% of repolarization (APD90) safety marker in absence and presence of drug simulated in a population of 110 hiPSC-CMs by STrhiPS.

We applied QT/TdP Risk Screen and STrhiPS to eight different drugs with known cardiac risk at the therapeutic drug concentration, C_max_ (Fig. 2a). For each compound, cardiac channel data for hERG, hCav1.2, and peak/late hNav1.5 were manually obtained at the Drug Safety Testing Center Co., Ltd. We provided these data as input to run each product according to their requisites. QT/TdP Risk Screen was used to predict the torsadogenic clinical risk of each compound. STrhiPS was applied to simulate experiments on a population of 110 hiPSC-CMs. Action potential duration at 90% of repolarization (APD_90_) values were estimated in absence and presence of the drug, and drug-induced repolarization abnormalities were automatically detected.

According to the CredibleMeds classification, all drugs are class 1 (unsafe) except for ranolazine (class 3—probably safe) and verapamil (class 4—safe). All drugs were correctly classified by QT/TdP Risk Screen except for ranolazine which was misclassified as class 1—unsafe (Fig. 2a). Misclassification of ranolazine by QT/TdP Risk Screen is not unique to this case study employing newly derived experimental data, but was also observed with literature-derived IC50 and Hill coefficient values and has been thoroughly discussed elsewhere.^18^

STrhiPS predictions of drug effects on the safety biomarker APD_90_ were in line with the CiPA and CredibleMeds TdP classifications: APD_90_ was prolonged in all unsafe drugs—particularly in the high-risk drug bepridil and flecainide—and it was shortened in the low-risk drug verapamil (Fig. 2b). For ranolazine some prolongation was estimated, likely owing to some difference between the Hill parameters obtained in this work and the ones found in literature.^18^ In our simulations, cells abnormalities were rarely observed at therapeutic concentrations (< 6% of cells in the population).

The two tools performed well individually. When used in combination they provided complementing information and strengthen each other’s outcomes in line with CiPA recommendations.

### Virtual Representation of a Self-expandable Heart Valve

Heart valve disease is prevalent throughout the world, and the number of heart valve replacements is expected to increase rapidly in the coming years. The implantation of transcatheter heart valves provides safe and minimally invasive means for heart valve replacement.^13^ Self-expandable valves (generally composed by a bioprosthetic valve sutured on a Nitinol stent) have the capacity of self-expanding when released from the catheter, enabling them to be fully repositionable and recapturable to optimize valve positioning before full release.

Computational M&S can help medical device companies to lower costs of multiple prototypes tests before manufacturing and collect scientific evidence for regulatory submissions.

#### SEV Radial Force Test

SEV (Self-Expandable Valve) Radial Force Test (sevradial.insilicocardio.com) is a web-based implementation of a new computational non-linear implicit finite element model that predicts the Radial Resistive Force and Chronic Outward Force in self-expandable heart valves, reproducing the ISO 5840-3 standard testing.^14^ The input requirements are the device mesh, nitinol super elastic mechanical properties and the minimum crimping diameter value to be tested. The tool can be used to test different prototype designs before manufacturing, or to test the final device design for regulatory submission, as a report following FDA guidelines^9^ is automatically generated.

##### *In silico* Testing of a Commercial Transcatheter Aortic Valve

**Figure 3:**
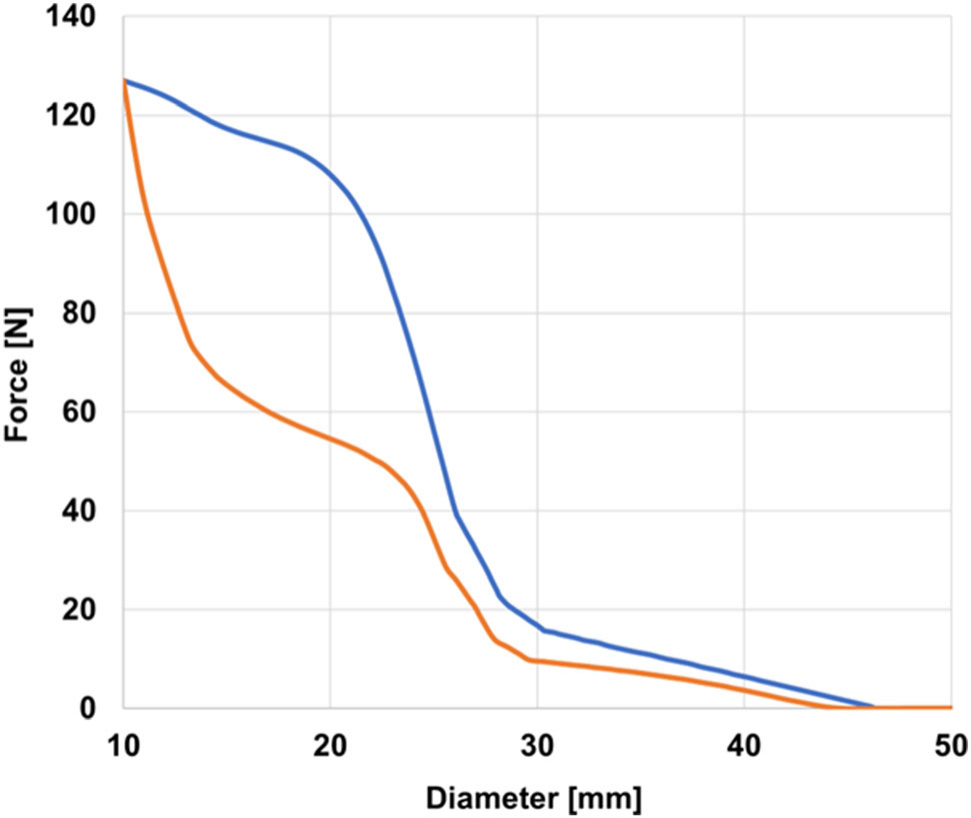
SEV Radial Force Test simulation results of the characterization of device radial force. Radial force as a function of the crimping diameter for a model representing a Corevalve 29 mm with crimping diameter equal to 10 mm. The blue and orange lines represent respectively the Radial Resistive Force, exerted during the radial compression, and Chronic Outward Force, exerted during the radial expansion, as a function of the diameter.

We applied SEV Radial Force Test to a 3D model of a SEV resembling the Corevalve 29 mm (Medtronic, Minneapolis, MN, USA).^5^ Nitinol material parameters were adopted from literature^12^ and a minimum crimping diameter of 10 mm was considered. Radial Resistive Force and Chronic Outward Force in function of the valve diameter were predicted (Fig. 3). The curves show the typical hysteretic behavior of Nitinol throughout a loading–unloading cycle, with higher force exerted by the device in correspondence with the imposed crimping diameter. Results show similarities to those found in literature for the same commercial product,^12^ with discrepancies in terms of maximum force related to the adoption of different modeling and meshing techniques, finite element solvers, and slight differences in the simulated SEV geometry and material. In addition to International Organization for Standardization (ISO) standard requirements, the tool allows to inspect strain values within the valve geometry through color maps of maximum principal strains.

### *In silico* Trial with Virtual Multiple Sclerosis Patients

Relapsing–remitting Multiple Sclerosis (RRMS) is characterized by stochastic attacks of the auto-reactive immune system on the body’s own myelin sheaths, damaging the central nervous system and causing the characteristic relapses and remissions. Patients affected by RRMS have dramatically different disease courses, necessitating a wide variety of treatment options with unique efficacy and safety profiles. Clinical trials to develop such treatments are necessarily expensive and long running as a result of RRMS’s generally slowly moving, unpredictable course. Recently, RRMS clinical trial costs have increased further^39^ as the current preference of active comparator over placebo and the substantial fall of relapse rates over the past few decades have led to a need for longer trials and larger groups to retain statistical power and the ability to observe treatment effects. Virtual patients can help reduce trial design by providing an *in silico* environment in which the (cost) effectiveness of the trial can be optimized.

#### MS TreatSim

MS TreatSim (Multiple Sclerosis Treatment Simulator, mstreat.insiliconeuro.com) is a web-based RRMS simulator, where virtual patients are personalized instances of a mechanistic, agent-based model,^23^ that emulates the immune system and the auto-immune response that characterizes RRMS and includes the option of the commonly prescribed treatment options interferon-β1a, teriflunomide, natalizumab and ocrelizumab. MS TreatSim can be set up by the user to simulate a clinical trial design, requiring input of population characteristics, selection criteria, trial groups and treatments, and trial timelines and milestones.

#### *In silico* Recreation of an Historical Trial of Natalizumab

In this use case, MS TreatSim was applied to recreate a historical phase III trial of the RRMS treatment natalizumab. In the AFFIRM clinical trial for natalizumab,^27^ the treatment group consisted of 627 patients receiving 300 mg of natalizumab every 4 weeks, and the control group was a placebo group (315 patients).

Due to lack of raw trial data availability, the recreation focused on recreating the global characteristics of the trial. The trial population was defined as follows: the age of onset distribution was set to 49.4%:36.2%:14.4% (18–29, 30–39 and 40–49 years), lesion load was set to high and oligoclonal bands status to present for all virtual patients, simulation duration prior to the trial was set to 5 years. Patients inclusion criteria were selected in order that patients were only included if they had experienced at least one relapse in the year preceding inclusion, but not in the final month. Two patients groups were created, mirroring the setup of the historical trial by maintaining a treatment: control ratio of 2:1. The natalizumab group (*n* = 80) was treated *in silico* with 300 mg of natalizumab every 4 weeks for 104 weeks, while the control group (*n* = 40) remained treatment naïve. Simulated time-to-relapse were analyzed using the Kaplan–Meier estimator with 95% Greenwood confidence intervals.

**Figure 4:**
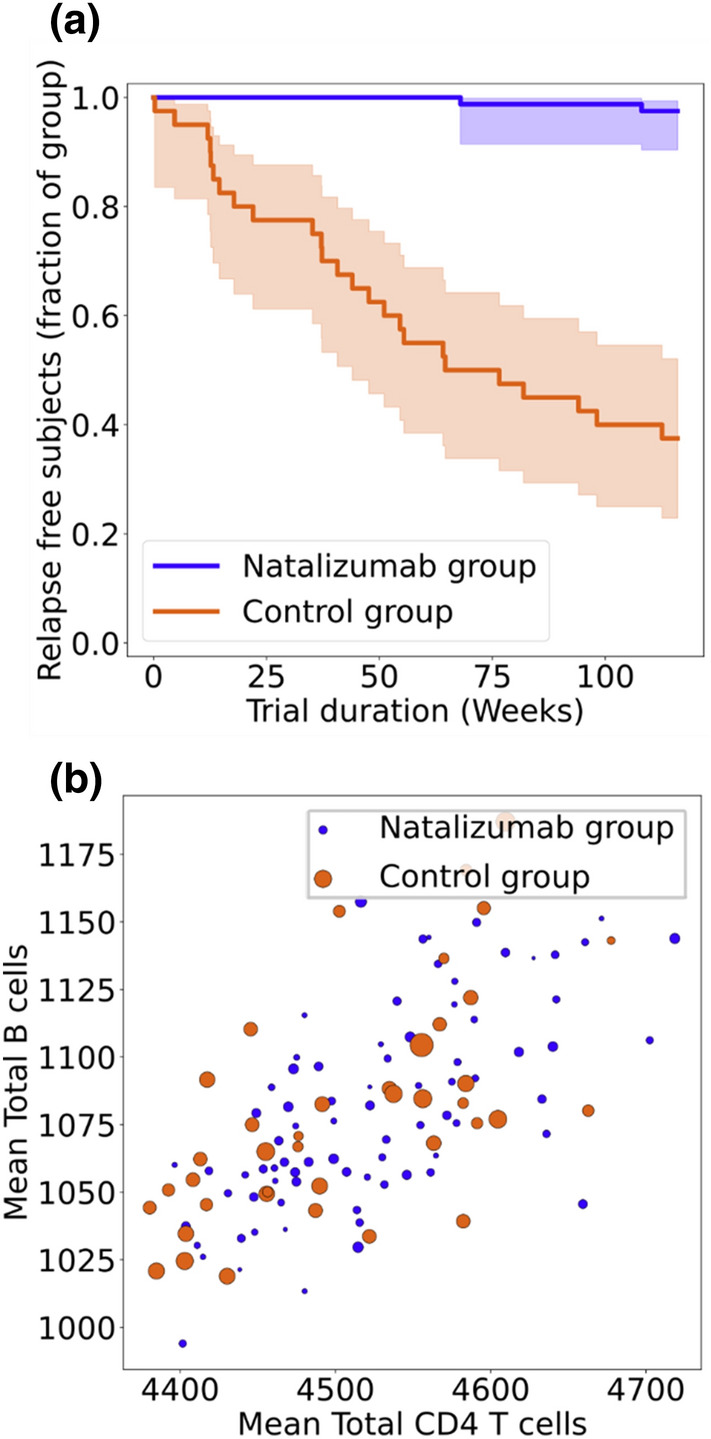
MS TreatSim results of the recreation of a natalizumab historical trial. (a) Kaplan–Meier curves of the relapse free patients in the natalizumab group and control group. Shaded areas denote 95% confidence intervals. (b) Bubble plot of total number of B cells (*y*-axis) vs. total number of CD4 T cells (*x*-axis) concentrations averaged over the entire trial duration. Markers represent individual virtual patients; markers sizes are scaled by the individual’s relapse rate, so that large markers denote patients with high relapse rates.

The *in silico* recreation of the historical trial with MS TreatSim generated full simulations of all virtual patients, which can be characterized at the trial level (Fig. 4a), or examined at the individual level (see e.g., Fig. 4b). In the simulated control group, the percentage of relapse free subjects after 104 weeks was estimated to be 40% (95% CI 25–55%) which very well included the historical trial relapse free subjects outcome of 41%. In the treatment group, natalizumab treatment effect was clearly reproduced with virtual patients showing substantially fewer relapses (over 90% of relapse free subjects at weeks 104) than in the control group. In comparison with the observed outcome of the historical trial (67% relapse free subjects at week 104), however, the simulation overestimated the natalizumab treatment effect. One possible explanation for this result may reside into the framework’s limitations to fully account for complex disease histories, which are prone to induce treatment resistance.^25^ This is supported by the observation that other metrics in the AFFIRM trial, as well as real world data on the effectiveness of natalizumab all support a larger treatment effect than that expressed in relapse free subjects alone.^25^

### Treatment Effects of a GnRH Agonist on a Virtual Cohort of Prostate Cancer Patients

PCa is one of the most common malignant tumors in men worldwide and the second leading cause of cancer related deaths in US men.^34^ Androgen deprivation therapy, achieved either by surgical or chemical castration, is the first line treatment against advanced prostate cancer and is also used to treat high-risk disease in combination with local radiation.^30^ Androgen blockade can be achieved through the administration of gonadotropin-releasing hormone (GnRH) agonists.^30^

Because treatment of prostate cancer with GnRH agonists requires chronic administration, administration of these drugs is usually performed in the form of sustained-release formulations. In this context, *in silico* clinical trials can help to optimize and investigate the PK profile of new sustained-release formulations that allow simplified dosing regimens, thus enhancing therapeutic compliance of patients and, consequently, their quality of life.

#### PCa GnRH Agonists Simulator

PCa GnRH Agonists Simulator (gnrhagonists.insilicoonco.com) is a web-based, cloud-based tool that allows to simulate the effects of GnRH agonists on a virtual population of prostate cancer patients. The tool is based on a PK/PD model that describes the suppression effect of testosterone after the administration of a GnRH agonist,^29^ simulates the drug and testosterone concentration levels in a population of virtual subjects and estimates the percentage of virtual subjects for whom testosterone levels are reduced below the castration limit during a trial.

Input requirements are the PK and PD properties of the drug, and the trial design. A library of sustained release formulations of known GnRH agonists is available including their PK and PD properties. Additionally, the tool can be customized for a new compound or drug of interest. In this case, the user is asked to setup a compartmental model to describe the PK of the drug. For the PD model, only the *in vitro* ligand–receptor equilibrium dissociation constant (*K*_d_) of the GnRH agonist is required.

##### *In silico* Recreation of the Virtual Cohorts Treated with Different Sustained-Release Formulations of Leuprolide

Leuprolide acetate is a GnRH agonist and it is commonly used in the treatment of prostate cancer.^30^ In this use case, PCa GnRH Agonists Simulator was applied to three cohorts from different leuprolide clinical trials. Description of the cohorts is provided in Table 1.

**Table 1:** Description of the three cohorts considered in this study. Leuprolide acetate was administered according to three different schedules.

Schedule name	Clinical trial reference	Number of patients	Schedule (dose/interval)	Study duration (days)
3M leuprolide	NCT01415960^33^	163	22.5 mg/3 months	168
4M leuprolide	M93-013^31^	45	30.0 mg/4 months	224
6M leuprolide	NCT00626431^35^	148	45.0 mg/6 months	336

For each schedule, leuprolide was administered twice (interval = interval between two schedules). The primary efficacy endpoint was the portion of patients achieving and maintaining the serum testosterone concentrations under the castration limit from day 28 to the end of the study

To replicate *in silico* the three cohorts, the three trial schedules (3M, 4M, and 6M) were simulated on a virtual population of 1000 prostate cancer patients. PK and PD properties were taken from the PCa GnRH Agonists Simulator library.^17^ Testosterone concentration castration limit was set to 0.5 ng/mL, as done in the real clinical trials. The study duration of each cohort is defined in Table 1.

The primary efficacy endpoint of the *in silico* clinical trial was the portion of patients achieving and maintaining the serum testosterone concentrations under the castration limit (0.5 ng/mL) from day 28 to the end of the study. The percentage and 95% confidence intervals of subjects achieving chemical castration was computed using the Kaplan–Meier method for right-censored observations.

**Figure 5:**
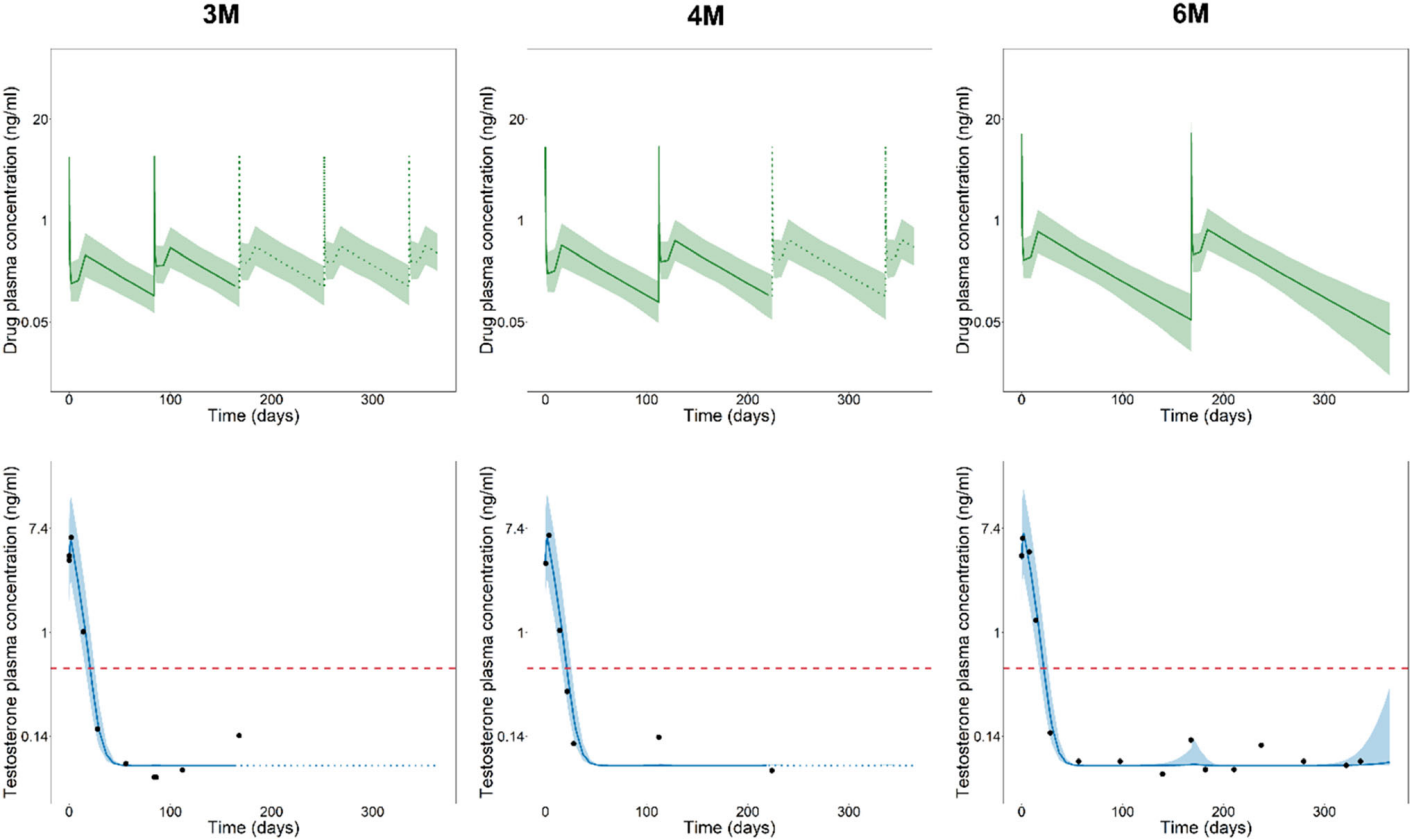
Results of the leuprolide cohorts recreation using PCa GnRH Agonists Simulator. Visual predictive checks of the drug plasma concentration (top row) and testosterone plasma concentration (bottom row) of the three schedules (3M leuprolide, 4M leuprolide and 6M leuprolide in the first, second and last column, respectively). Continuous lines represent the median simulated profiles in the time frame where real clinical data are available; dotted lines are the median of the simulated profiles in the time frame where clinical data are not available; shaded areas represent the 95% prediction intervals obtained from 1000 individual simulated profiles. In the bottom row, black dots represent the experimental mean testosterone levels in plasma from previous studies. The red dashed line represents the testosterone concentration castration limit (0.5 ng/mL). 3M leuprolide = 22 mg leuprolide injected once every 3 months; 4M leuprolide = 30 mg leuprolide injected once every 4 months; 6M leuprolide = 45 mg leuprolide injected once every 6 months.

In Fig. 5, observed and simulated data for the three cohorts are displayed. Observed data were extracted from publications.^31,33,35^ Results of the *in silico* clinical trials were in good agreement with the outcomes of the actual trials. Visual predictive checks of the testosterone concentrations in plasma well contained the mean testosterone levels measured in actual patients. The percentages of virtual and real patients^31,33,35^ achieving and maintaining the testosterone levels below 0.5 ng/mL are summarized in Table 2.

**Table 2:** Percentage of virtual patients (third column) achieving and maintaining chemical castration from day 28 to the end of the study (168, 224 and 336 days for 3M, 4M and 6M, respectively) computed with the Kaplan–Meier method and percentage of real patient achieving chemical castration.

Schedule	Estimate of the virtual population (95% CI)	Estimate of the real population	Relative error (%)
3M leuprolide	99.8% (99.5%, 100%)	96.8%^33^	3.0
4M leuprolide	99.6% (99.2%, 100%)	94.0%^31^	5.9
6M leuprolide	98.3% (97.5%, 99.1%)	93.4%^35^	4.9

The relative prediction error was computed as the difference between the estimates of the virtual population and the real population over the estimate of the real population. CI = confidence interval. 3M leuprolide = 22.5 mg leuprolide injected once every 3 months; 4M leuprolide = 30 mg leuprolide injected once every 4 months; 6M leuprolide = 45 mg leuprolide injected once every 6 months

*In silico* clinical trials using the cloud-based tool PCa GnRH Agonists Simulator can reliably reproduce testosterone suppression in prostate cancer patients. The results of this use case obtained in few minutes and with a mean prediction error over the three schedules equal to 4.8% further corroborate this conclusion.

## Discussion

Recent analyses estimated research and development investment to bring a new compound to the market to be $1.3 billion on average, with costs depending on therapeutic areas, ranging from $765.9 million for nervous system agents to $2771.6 million for antineoplastic and immunomodulating agents.^38^ Similarly, in 2010 it was estimated that the average total cost to bring a new medical device from concept to approval in US varies from approximately $31 million for a low-to-moderate-risk 510(k) product, up to $94 million for a higher-risk PMA product.^19^

The use of *in silico* trials in the development or regulatory evaluation of new treatments is meeting expectation of beneficial results in terms of cost and time to market reduction.^6^ Indeed, *in silico* trials provide unique data that may be used to: predict performance in the short and mid-long term and avoid failures in development; quickly evaluate the potential applicability of a medical product in various clinical scenarios; evaluate adverse events without risk to animals or patients.^36^ This paper presented two preclinical use cases and two clinical use cases of *in silico* trials performed via simulation tools available on the InSilicoTrials.com platform.

Traditionally, the preclinical phase of a drug development program evaluates the toxicity and efficacy of a new compound by means of *in vitro* and *in vivo* experiments. The first use case illustrates how *in silico* trial methodologies based on virtual human adult and fetal cardiomyocyte cells models can support the preclinical evaluation of drug-induced cardiotoxicity by leveraging *in vitro* experimental data to i) anticipate torsadogenic clinical risk-outcomes (QT/TdP Risk Screen) and ii) reduce the number of time-consuming and expensive *in vitro* tests on hiPSC-CMs (STrhiPS), while at the same time improving the predictive potential of *in vitro* results.

For medical devices, standard tests performed during preclinical phases are used to evaluate device safety. The second use case shows how *in silico* methodologies can support the characterization of the mechanical behavior of a cardiovascular device, providing prediction comparable to literature results. The SEV Radial Force Test application allows for *in silico* reproduction of bench tests to virtually assess a new medical device design, and to identify the worst-case within a series of different prototypes to reduce and refine the physical test burden in terms of time and costs.

The clinical phase of a drug development program evaluates the toxicity and efficacy of a new compound by means of clinical studies. The MS TreatSim application presented in the third use case simulates realistic virtual patients and clinical trials, broadening the original scope of this model from individual applications^23^ to population-level simulations. Such population-level simulations can be leveraged throughout the clinical development of a new drug and beyond, with applications including: the design and de-risking of clinical trials, the selection of sub-populations that will respond best to a new therapy, the augmentation or even replacement of control arms in phase II and III studies, the evaluation of efficacy and safety in long-term scenarios to support label extension requests or post-marketing trials. In addition, the mechanistic nature of the model integrating a detailed and comprehensive description of the immune system is a key feature of the tool and provides opportunities for several applications. For example, patient specific simulations could be applied for individual prognosis and treatment planning, whereas incorporation of mechanism of action for new or existing drugs could be used to explore novel therapies and combination strategies.

In the same vein, the PCa GnRH Agonists Simulator illustrated in the fourth use case can help to optimize the design of a clinical study by identifying the most promising drug formulations and dosing regimens, allowing to suppress testosterone levels below castration levels in most prostate cancer patients.

Every computational approach is suited to fulfill a given objective. Bottom-up, mechanistic models perform very well for hypothesis testing and are adaptable, allowing to incorporate additional elements. When properly validated, these models may provide useful predictions beyond the data employed for model building. For example, mechanistic PBPK simulations are now an industry standard and accepted by regulators in replacement of real clinical studies for the evaluation of drug-drug interactions.^32^

To deal with always increasing amounts of data that could be integrated in mechanistic models to improve their predictions, there is great potential in combining mechanistic modeling approaches with machine learning algorithms, described by the term “mechanistic learning”.^3^ An example of such modeling methodology is provided in the first use case presented in this paper. Indeed, the QT/TdP Risk Screen classifier derives *in silico* biomarkers from simulations of a mechanistic cellular cardiomyocyte model and utilizes them as inputs of a machine learning classifier algorithm.

Computational M&S is thus an invaluable asset in the healthcare industry. However, the need for advanced know-how and computational resources restricts its adoption mainly to large biotechnology and pharmaceutical companies. Making M&S available to a broad spectrum of potential users (medical device and pharmaceutical companies, hospitals, healthcare institutions) would require an easy and controlled access to M&S resources in a secure environment. Furthermore, a joint effort between academia, industry and regulatory bodies is needed to reach a rapid adoption of such a harmonized approach. The InSilicoTrials.com platform aims to disrupt the healthcare landscape, lowering entry barriers for investment and high-level knowledge requirements. It provides healthcare companies and researchers with integrated and easy-to-use simulation workflows to perform computational testing during the development and validation process of new medical devices (e.g., orthopedics, cardiovascular, wearable) and drugs (e.g., PK/PD, disease progression, QSP). Furthermore, the platform is currently being expanded to integrate additional functionalities, including clinical data storage and sharing, medical image analysis and segmentation tools, and access to virtual patients in different disease areas, enabling performing design of experiments, parametric and optimization studies. Based on a web-based collaboration paradigm, the platform represents a cutting-edge solution for the life science and healthcare environment, finally resulting in an increased pace of innovation and reduced time-to-market for new treatments.
